# Enhancing Bandwidth of Cantilever-Based Energy Harvester Using a Passive Multi-Chamber Movable Mass Repositioning Mechanism

**DOI:** 10.3390/mi17070849

**Published:** 2026-07-17

**Authors:** Nico E. Galarza, Nathan Jackson

**Affiliations:** 1 Department of Electrical and Computer Engineering, University of New Mexico, Albuquerque, NM 87131, USA; ngalarza1@unm.edu; 2 Center for High Technology Materials, University of New Mexico, Albuquerque, NM 87106, USA; 3 Department of Mechanical Engineering, University of New Mexico, Albuquerque, NM 87131, USA

**Keywords:** energy harvesting, bandwidth, cantilever, piezoelectric, movable mass, frequency, power

## Abstract

Piezoelectric energy harvesters (PEHs) have emerged as a promising solution for self-powered small-scale electronic systems; however, their narrow operational bandwidth limits performance under varying excitation conditions commonly found in ambient environments. To address this limitation, this study proposes a passive multi-chamber proof-mass design containing internal free-moving masses to enhance the frequency bandwidth of a PEH through nonlinear dynamics. Multiple proof-mass models were designed and experimentally evaluated using tungsten and Teflon spherical movable masses under 0.5 g and 1 g excitation levels. Design alterations include varying the number of chambers, which in essence reduces the maximum lateral displacement of the movable mass. Baseline characterization was first conducted using empty proof-mass configurations, followed by fixed-mass and free-mass testing to isolate the effects of dynamic mass repositioning. The results demonstrate that baseline and fixed-mass configurations remained limited to bandwidths in the 5–8 Hz range. In contrast, free-mass configurations produced significant bandwidth enhancement across all models. The largest bandwidth increase consisted of a multiple-chamber proof-mass design, which resulted in a bandwidth of 72 Hz using tungsten rolling spheres at 1 g excitation, corresponding to a 1400% increase relative to the baseline conditions.

## 1. Introduction

Piezoelectric energy harvesters (PEHs) have emerged as a promising solution for self-powered small-scale electronic systems, particularly in applications such as wireless sensor networks (WSNs) and remote monitoring devices where battery replacement would be impractical [1]. WSNs are one of the main components used in smart buildings to assist health monitoring and supplying data on critical applications. PEHs typically utilize ambient vibrations as an energy source, which are widely available across environments such as transportation systems and structural infrastructure. PEHs can offer consistent energy harvesting capabilities as vibrations continue [2]. PEHs can also be used in various applications such as machine fault diagnosis for machines or trains [3,4].

PEH devices are commonly designed in the form of a cantilever beam structure, converting mechanical vibrations into electrical energy through strain-induced charge generation [5,6,7,8]. There have been numerous attempts to increase power density via optimization of cantilever shape [7,9], piezoelectric materials [10], electrode design [11,12,13] and various other methods. However, their performance is strongly dependent on maximizing strain/stress in the cantilever, which is achieved when the cantilever operates at resonant frequency, thus obtaining maximum power output. However, a major limitation of these systems is that most cantilever structures have a narrow operational bandwidth, which hinders their performance under varying or broadband excitation conditions commonly found in real-world environments and applications [14,15]. Silicon-based Microelectromechanical System (MEMS) PEHs have even lower bandwidths (1–3 Hz or ~1% of resonant frequency), which limits their use in commercial applications [16].

To address this limitation, a wide range of bandwidth-enhancing methods have been previously explored. The integration of non-linearities have been studied, including Duffing resonators and bistable systems, which shift the resonance frequency (RF) and enable broadband responses through nonlinear stiffness effects [14,17,18]. Hybrid nonlinear approaches combining bi-stability and internal resonance have further demonstrated improved frequency response over a wider range [15]. While these methods can significantly enhance bandwidth, they often introduce unpredictable and complex dynamics that have also shown reduction in power production and stability. Other methods include active tuning mechanisms that alter the proof mass [19,20,21,22].

Impact-based mechanisms have also been investigated as a means of inducing broadband performance. Mechanical stoppers and vibro-impact setups introduce linear dynamics that can extend the frequency response range and improve energy harvesting performance [23,24,25,26]. These approaches rely on controlled collisions within the system to redistribute energy across a wider range of frequencies, though they may introduce durability concerns and increased mechanical wear over time.

Another promising direction involves the use of movable or reconfigurable masses to dynamically alter the system’s physical properties. Existing work on movable mass-based designs have shown significant bandwidth enhancement by introducing center-of- gravity (CoG) shifts and inertial effects using a rolling mass [27,28]. Vertical jumping masses have also been investigated, which focus on changing the effective mass due to free-fall moving masses, which increased the bandwidth considerably compared to fixed-mass systems, primarily due to continuous modulation of the system’s effective mass, thus shifting its RF [29]. Similarly, liquid-based movable masses, including ferrofluid and liquid metal embedded masses, have achieved substantial bandwidth widening by enabling fluid-induced CoG shifts and nonlinear damping effects [30,31,32,33,34,35,36]. These methods provide a passive means of tuning, which results in a broadband performance, although fluid dynamics can introduce additional complexity and variability due to uncertainties and possible packaging issues.

Magnetic-based tuning and nonlinear dynamics have also been explored to achieve bandwidth expansion. Devices integrating permanent magnets to enable bidirectional tuning of the resonant frequency can enhance both bandwidth and power output depending on the magnets configuration [37,38,39,40]. However, integrating magnetic components into MEMS scale systems can present fabrication challenges and may increase system complexity.

More recently, combined excitation and multi-modal energy harvesting approaches have been explored, such as systems capable of simultaneously harvesting wind and vibrational energy or operating under off-resonance conditions [26,41]. These designs focus on improving energy capture under real operating conditions but often require more complex system designs.

Despite these advancements, many existing approaches are limited to single-degree-of-freedom motion or the inability to simultaneously exploit multiple nonlinear mechanisms within a single structure. In particular, while movable mass systems have demonstrated strong potential for bandwidth enhancement, their integration is often constrained to simple geometries that do not fully leverage multi-directional dynamics.

The research in this paper is based on an initial results published in a conference paper, which illustrated potential enhancement in bandwidth using a two-chamber system [42]. In this work, multi-chamber proof-mass designs were proposed to enhance the bandwidth of a piezoelectric cantilever energy harvester through internal free-moving masses. The proposed design incorporates multiple internal chambers (1C–4C models) containing spherical movable masses that enable both lateral motion and constrained vertical displacement. This dual motion introduces parallel nonlinear effects, including CoG shifting, variable mass, and impact dynamics, resulting in a significantly broadened frequency response. The objective of this study is to experimentally evaluate the influence of multi-chambered proof-mass configurations, which limit the CoG change by confining the lateral displacement of the movable mass, thus optimizing bandwidth of a PEH. The work presented in this paper focuses on a macro-scale version to investigate the optimization, but the concept of using movable masses could potentially be scaled down to the MEMS scale in the future. Baseline characterization was first conducted using empty proof-mass configurations, followed by fixed-mass integration and finally free-mass configurations to isolate the effects of varying mass distribution and nonlinear dynamics. A comparison study of the various proof masses was investigated to determine which configuration was optimal for increasing bandwidth.

## 2. Materials and Methods

**Figure 1:**
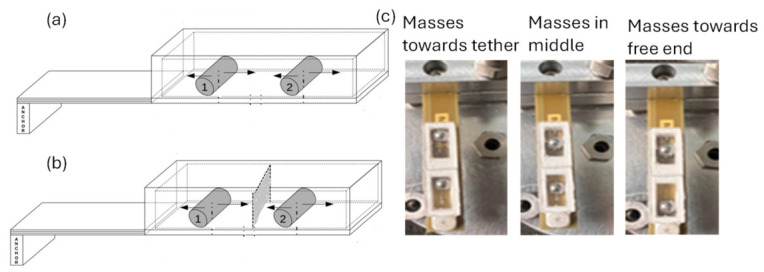
Overall concept for (**a**) a single chamber with movable mass inside the proof mass, (**b**) a 2-chamber concept of the movable mass, and (**c**) image of a 2-chamber proof mass with tungsten movable mass illustrating various positions of the movable masses as the cantilever deflects.

**Figure 2:**
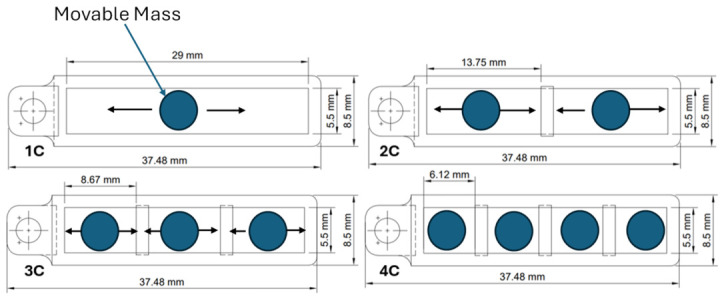
Top-view schematic of the four proof masses with different number of chambers with dimensions and the circular mass indicating movement of the spheres.

This work utilized a commercially available piezoelectric cantilever model (S129-H5FR-1803YB, Mide Technology, Woburn, MA, USA), consisting of multiple supporting layers bonded to a lead zirconate titanate (PZT) piezoelectric ceramic layer with a d_33_ value of 650 pC/N. The device measured 71 mm in length, 10.3 mm in width, and 0.74 mm in thickness, and these dimensions served as the reference for the proof-mass models developed during this work. The testing equipment consisted of a Labworks ET126-3 electrodynamic vibration shaker (Costa Mesa, CA, USA) driven by a linear power amplifier. The device under testing was securely clamped to the shaker using a custom-made clamp and connected to an Agilent SDS 2102-X oscilloscope to record open-circuit frequency response of the device. All measurements were the averages of the peak-to-peak voltages (V_pp_) at a specific frequencies over a 5 s window. The overall concept of the movable mass proof-mass system is shown in Figure 1 and Figure 2.

### 2.1. Multi-Chambered Proof-Mass Model Designs

Multiple proof-mass models were designed and 3D printed using polylactic acid (PLA) during this work. These include the models 1C, 2C, 3C, and 4C, where the number included in the model’s name represents the total number of internal chambers (C). All models consist of the same base dimensions; however, as mentioned, the number of chambers and overall internal length of the chambers vary according to the model as shown in Figure 2, where 2C has an internal chamber length of 13.75 mm per chamber while 3C has 8.67 mm per chamber. The diameter of the movable mass was approximately 5 mm. The width of the chamber was set at 5.5 mm throughout the various designs to allow the movable mass to roll along the length of the cantilever or chamber but preventing it from moving in the lateral direction. Therefore, the effective overall movable length was 24 mm, 8.75 mm, 3.67 mm, and 1.12 mm for 1C, 2C, 3C, and 4C respectively as shown in Table 1. The proposed movable masses consisted of tungsten or Teflon 5 mm spheres. The two materials were chosen due to their large difference in density. The tungsten spheres should provide a larger CoG change due to the increase in overall mass. Teflon was chosen as it should be able to move with lower acceleration as the lower density and low friction will require less energy to cause movement in the spheres. This is based on previous results which demonstrates Teflon spheres enhanced bandwidth at lower accelerations compared to denser movable masses [29]. Another key parameter to consider is the total mass of the proof mass itself, which consists of the 3D printed proof mass (given in Table 1) and the additional mass of the movable sphere. The movable mass changes for the various designs as the number of spheres can vary between 1 and 4. The overall mass and the location of the CoG are critical for determining the RF as illustrated in Equations (1) and (2).

Note that the mass values from Table 1 represent the empty proof masses without the PEH or the plastic M3 nut and bolt used to secure the assembly. Therefore, to calculate the total assembly mass, the cantilever’s mass, being 1930 mg and the M3 screw and nut’s mass being 280 mg, should be considered. This is an important consideration for the overall design since increased mass lowers the theoretical RF of the cantilever, which is represented by the simplified Equation (1), where E is the elastic modulus of the beam, *m* is the proof mass, and *w*, *t*, *L* are the width, thickness and length [29].

(1) $$f=\frac{1}{2\pi }\sqrt{\frac{3E}{m}}\sqrt{\frac{wt^{3}}{L^{3}}}$$

The RF is also influenced by the position of the CoG of the proof mass, which can be expressed as:

(2) $$f^{\prime }=\frac{1}{2\pi }\sqrt{\frac{Ewt^{3}}{12mL^{3}}\times \frac{r^{2}+6r+2}{8r^{4}+14r^{3}+10.5r^{2}+4r+\frac{2}{3}}}$$

where “*r*” represents the normalized position of the CoG along the length of the cantilever. As the device experiences oscillatory deflection, the internal movable masses shift within the chambers, dynamically altering the CoG and, consequently, the effective resonant frequency of the system.

The CoG is important because this initial RF calculation represents a fixed limiting point in the device’s possible operating range. When the mass consists of a movable component the CoG changes and thus the RF will change for that specific instant in time. Thus, the movable masses cause the device to go in and out of RF thus widening the bandwidth. Chambers that have more movable masses will have a lower RF due to the overall mass being higher thus reducing the RF. Once the movable mass is added to the system, both the vertical and horizontal movement of the mass is important. Since all proof-mass internal chambers are designed with a 5.25 mm height, a 0.25 mm gap exists once the 5 mm diameter spheres/masses are added. This height is not large enough to cause significant free-fall time, which would affect the overall effective mass. Instead, the small height gap causes small impacts which can impact power via applied stress, especially under high acceleration applications. Thus, when excited, the mass has room to both bounce and roll during the cantilevers bending cycle. The bouncing effect on the effective mass is limited due to the low gap height of 0.25 mm and thus the rolling phenomena will have the largest impact on altering the bandwidth. The impact of the bouncing sphere can cause some nonlinear dynamics but is primarily used to increase power by stressing the piezoelectric cantilever. The gap could be changed in future studies to implement an effective mass change. Parallel to this, the mass also has room to shift its position along the length of the cantilever, this changes the device’s effective CoG.

By combining two movable mass methods, it is possible to simultaneously shift two variables in Equation (2), which in theory should result in a more aggressive active shift in RF during testing. However, due to the small vertical gap of 0.25 mm in the internal chamber, the vertical movement’s contribution should be limited when compared to other vertical moving mass dedicated models as seen in other studies [29]. The main motivation for including the vertical gap in the design was to allow the mass to reposition along the length of the cantilever without any major obstructions.

### 2.2. Fixed-Mass Configuration Testing

**Figure 3:**
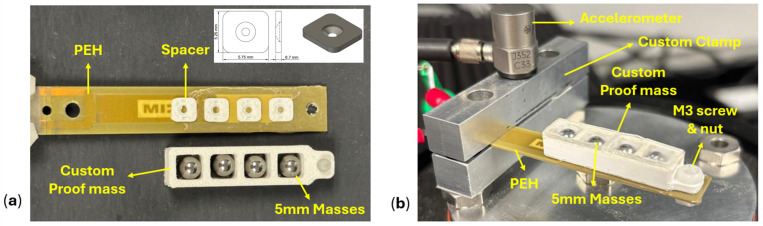
Image of the energy harvesting system (**a**) top view illustrating the fixed spacers and proof mass with a 4-chamber proof mass (inset shows schematic of spacer dimensions) and (**b**) an image of system on the vibration shaker.

To assess the difference in performance of the device associated with free-moving vs. fixed-mass configurations, a reliable and repeatable method for securing the movable masses inside the chambers of the proof mass was required. Adding any masses will affect the RF, so ideally, we needed a method to hold the spherical masses in place to compare the power output for the various devices. Having these requirements in mind, Figure 3 shows the proposed 3D printed spacer that was developed for this work. Said spacer was printed using PLA, and the dimensions were: 5.25 mm length, 5.75 mm width, and a thickness of 0.6 mm. The mass of the spacer was measured to be approximately 0.021 g, therefore being close to negligible relative to the proof mass overall mass of 1253 mg for the heaviest 4C and 1078 mg for the lightest case 1C.

A total of 4 spacers were printed to hold up to four masses at a time, a necessity for model 4C which features a four-chamber system. Figure 3 demonstrates how the spacers were secured using a thin layer of double-sided adhesive placed on the surface of the PEH in such a way that the said spacers align with the proof-mass chambers when assembled. A similar strategy was employed for models 1C, 2C, and 3C. By integrating the spacer inside the chambers, the movable mass was pinched in place, rendering it fixed even under 1 g acceleration testing.

## 3. Results and Discussion

The performance of the proposed multi-chambered proof-mass designs were evaluated using controlled frequency sweep tests. Open-circuit voltage measurements were taken for both fixed- and free-mass configurations using tungsten and Teflon spheres across multiple proof-mass models (1C–4C) and excitation levels of 0.5 g and 1 g. Accelerations were varied to alter the cantilevers displacement thus increasing the mobility of the movable mass. The goal of these experiments was to assess the effects of mass distribution and different movable mass percentages on the PEH’s bandwidth.

To establish a reference that can serve for comparison, the baseline frequency response of the cantilever with an empty proof mass was first characterized. Following this, fixed masses were introduced to identify the effects of added mass without dynamic repositioning. Then passive movable mass configurations were evaluated to identify how they influence the device’s bandwidth. This approach allows for a direct comparison between a fixed resonant frequency associated with a fixed mass and the adaptive nonlinear response introduced by the multi-chambered free-mass configuration.

### 3.1. Initial Proof-Mass Characterization

**Figure 4:**
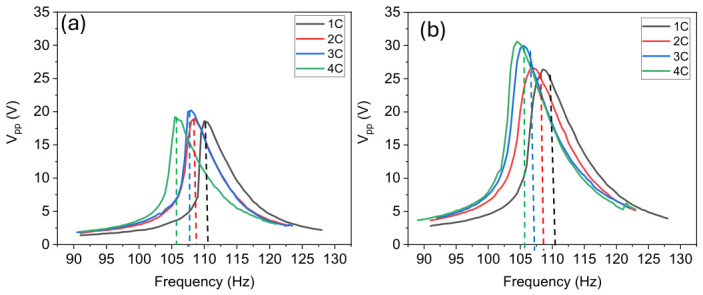
Empty proof mass (**a**) 0.5 g and (**b**) 1 g test results representing baseline performance for the proposed models dashed line shows the RF of each device.

The initial characterization of the proof-mass designs was conducted using empty proof-masses to establish a baseline response before the integration of movable masses. Frequency sweep tests were performed under controlled acceleration levels, which allowed us to measure the V_pp_, RF (based on highest V_pp_), and bandwidth (based on full-width half maximum (FWHM)) for the various proof-masses. This baseline assessment identifies the effects of integrating the proof-mass structure itself, ensuring that any additional changes in device’s performance can be associated with the addition and dynamic behavior of the movable masses. Figure 4 shows the frequency response of the empty proof-mass configurations for all proof mass models (1C–4C) under excitation levels of 0.5 g and 1 g. The measured FWHM at 0.5 g was found to be 5.6, 5.8, 5.6, and 5.8 Hz for the 1C, 2C, 3C, and 4C models, respectively. At 1 g, the bandwidth increased to 8.2, 8.3, 7.0, and 7.5 Hz for the same models. These values were similar to the base bandwidth of the cantilever itself, which was approximately 7 Hz under baseline conditions at 1 g. Therefore, these results demonstrate that no significant change in bandwidth was introduced by the proof mass models when tested without movable masses. Since the mass of the proof mass did not vary significantly (16% mass deviation between 1C and 4C) the RF was approximately the same. There was a slight shift to the left (lower frequency) as the number of chambers increased, which resulted from an increase in the overall mass. Voltage output increased as acceleration increased, which was also expected since the cantilevers would have higher strain/stress at higher acceleration. The figure shows a typical linear response.

Calculations provided in Table 2 show the calculated mass change after adding the spacers for each model and mass configuration. This calculation was performed to assess how the added spacer mass affects the total effective mass of each of the models. The expected mass shift calculation is based on the addition of the spacer. The added spacers cause minimal mass change thus they should not significantly affect the RF. However, adding tungsten or Teflon spheres has a larger impact on the mass change, and thus they will reduce the RF.

**Figure 5:**
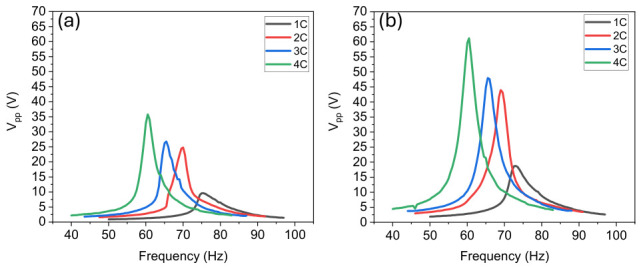
Output voltage as a function of frequency for fixed-masses (**a**) tungsten 0.5 g and (**b**) 1 g fixed mass configuration.

Throughout the different proof-mass models and mass configurations, the expected shift in effective mass due to the added mass of integrating the spacers was at its greatest 2.1% for the 4C model and 0.5% for the 1C model, due to adding four spacers (4C) vs. only one spacer (1C). The RF shift due to the spacers was minimal at approximately 1 Hz for the 4C proof mass with spacers as shown in Figure 4. The spacers and spherical masses were integrated into each mass model accordingly. The spacers fixed the masses, so they did not move, which was visually verified during the test. Frequency sweeps in 0.1 Hz intervals were performed (sweeps were performed in both directions), and V_pp_ was monitored. The results shown in Figure 5 demonstrate that the fixed tungsten mass configuration had a peak voltage of 35.8 and 61.2 V_pp_ for 0.5 and 1 g accelerations for the 4C proof mass. This illustrates a significant increase in output voltage, which was expected since the overall effective mass increased by approximately 405% due to the four tungsten spheres. Additionally, a bandwidth of 6, 4.5, 4.8, and 4.1 Hz for the 0.5 g acceleration test was achieved, while for the 1 g acceleration test resulted in a bandwidth of 8, 4.8, 5.1, and 4.9 Hz for 1C, 2C, 3C, and 4C models respectively. Adding fixed masses did not show any significant increase in bandwidth compared to the control (no tungsten or Teflon masses). However, the voltage output did increase due to the increased mass, which increased stress for the same acceleration. The 4C fixed mass system illustrated the highest power, as expected. The RF of the devices was also reduced as the overall mass increased. The output results also resemble a typical linear response due to the spheres being fixed in place along the length of the cantilever.

**Figure 6:**
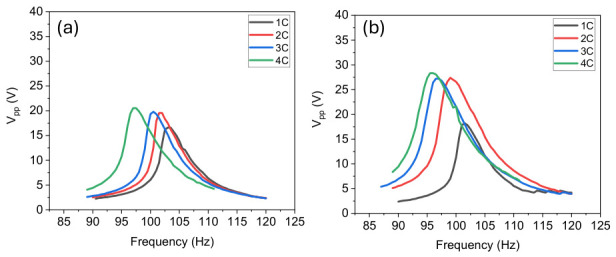
Output voltage as a function of frequency for fixed-masses with (**a**) Teflon 0.5 g and 1 g (**b**) fixed mass configuration test results.

Following this, the test was repeated using the Teflon masses with the same configuration for each model. The results shown in Figure 6 showed similar narrow bandwidths with little to no variation throughout the different mass configurations and accelerations. The bandwidth results were 6.1, 5.9, 6.2, and 6.5 Hz for the 0.5 g acceleration test, while the 1 g tests results were 8.33, 8.2, 8.45, and 8.1 Hz for 1C, 2C, 3C, and 4C models respectively. Since Teflon has a lower density than tungsten the overall mass was lower compared to system with tungsten, this resulted in a lower voltage output. The Teflon mass configuration systems did result in lowering the RF slightly compared to the control (no Teflon masses), and the voltage output also increased slightly. However, the change in RF and voltage were much lower than with tungsten which was expected due to the density difference.

### 3.2. Movable Mass Configuration Test Results

The spacers were removed for the movable mass configurations to allow the spherical masses to be free to move in the confinement of the chambers. This configuration allows the masses to passively adjust their position along the length of the cantilever during testing based on the deflection of the cantilever, since deflection will be largest towards the free end the masses at this location will likely have more influence than masses towards the fixed end. This repositioning allows the masses to alter the CoG of the device, thus shifting the effective RF during testing. Therefore, while the cantilever is oscillating it is going in and out of RF, which leads to reduction in peak voltage output as shown but increases bandwidth. Since the output was no longer a linear sinusoidal output the average V_pp_ values over a 5 s window were used for the output data at a specific frequency. The length of the chambers affects the total mass shift that was potentially available. The 4C configuration does not allow for significant rolling and is meant to mimic the fixed spacer configuration. The change in CoG caused by the movable masses can then increase the operating frequency range or bandwidth of the device as long as the cantilever undergoes enough deflection to cause the movable masses to shift in their position. This method of increasing bandwidth requires significant displacement of the movable masses, which can be enhanced by higher acceleration (increasing displacement of the cantilever) and lower frequency (as the masses need time to move). Therefore, this technique would not be as efficient for high frequency low acceleration applications. Previous studies show that the masses move towards the free end of the cantilever during downward bending and move towards the fixed end during upward bending of cantilever, along with some vertical displacement caused by the high acceleration applied during testing [27,28]. This nonlinear behavior results in an electrical output during frequencies that the device usually would not produce. This results in an overall larger operating frequency range or bandwidth. The voltage response is no longer a linear (single sinusoidal) response, as the cantilevers RF is constantly changing which leads to wider range of output responses.

**Figure 7:**
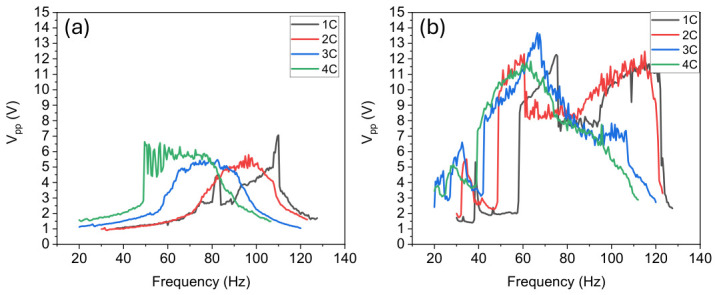
Experimental results of voltage output vs. frequency for movable masses with different chamber configurations for (**a**) tungsten 0.5 g and (**b**) 1 g free-mass configurations.

**Figure 8:**
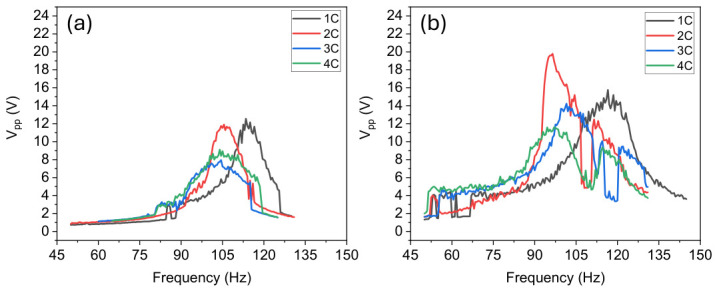
Experimental results of voltage output vs. frequency for movable masses with different chamber configurations for (**a**) Teflon 0.5 g and (**b**) 1 g free-mass configurations.

Figure 7 and Figure 8 illustrate the results for the tungsten (Figure 7) movable masses and the Teflon (Figure 8) movable masses. In both cases the voltage output was significantly increased for the 1 g acceleration compared to the 0.5 g. As illustrated, the voltage response was not linear with a single symmetric peak but instead a nonlinear output resulting in multiple peaks, which basically resembles randomized high peak noise. The bandwidth results are illustrated in Table 3. Table 3 summarizes the FWHM results for all proof-mass models (1C–4C) across baseline, fixed-mass, and free-mass configurations under 0.5 g and 1 g excitation levels. The baseline results, corresponding to the empty proof-mass configurations, remain within a narrow range of approximately 5.6–5.8 Hz at 0.5 g and 7.0–8.3 Hz at 1 g.

The fixed masses demonstrated slight variations in bandwidth across all proof-mass models. The FWHM values remained close to the devices baseline values, generally ranging between 4.1 and 6.5 Hz at 0.5 g and 4.8–8.45 Hz at 1 g. In several cases, particularly for tungsten, a slight reduction in bandwidth was observed. This behavior was attributed to the increase in effective mass, which shifts the resonant frequency but does not introduce the nonlinear dynamic effects required for bandwidth broadening. With the reduction in RF the bandwidth as a percentage of the RF was approximately the same. These results indicate that static mass additions alone were insufficient to significantly enhance the operational bandwidth of the device.

In contrast, the free-mass configurations produce a considerable increase in bandwidth across all proof-mass models. At 0.5 g, the bandwidth increased significantly, reaching up to 35.7 Hz for the 3C tungsten configuration and 31.1 Hz for the 2C tungsten configuration, compared to baseline values near 5–6 Hz. At 1 g, even greater enhancements were observed, with maximum bandwidths reaching 72 Hz for the 2C tungsten configuration. This represents a 14x (1400%) increase in bandwidth compared to the baseline condition. The 1C also demonstrated significant increase in bandwidth but the 3C and 4C showed a significant decrease in bandwidth compared to the 1C and 2C, which was believed to be due to the lack of displacement of the mass due to the confines of the chamber dimensions. The Teflon spheres demonstrated more consistent bandwidth values across all proof-mass configurations. The reasoning for this was that there was less movable mass due to the density limits, so the CoG change was about the same regardless of the amount of movement. In addition, the Teflon 1C at 0.5 g had a larger bandwidth than the tungsten because the sphere moved easily with lower acceleration, whereas the tungsten mass needed higher acceleration to cause more significant movement or change in CoG. Therefore, lower density movable masses do not cause as much bandwidth enhancement, but they can be a better option when operating at lower accelerations. The system also had other nonlinear effects other than CoG change from rolling, such as vertical impacts from bouncing, lateral impacts on the spacers between chambers, and friction which could be investigated in the future. The long-term effects of these impacts will also need to be investigated to determine if they damage the system over time.

The observed bandwidth enhancement can be associated with the introduction of nonlinear dynamic behavior originating from the motion of the internal masses. As the device oscillates, the movable masses cause continuous shifts in the CoG while also producing intermittent impact interactions within the chambers. These combined effects result in a wider frequency response, leading to the observed broadening of the bandwidth. Furthermore, increasing the excitation level from 0.5 g to 1 g amplifies these nonlinear effects, resulting in more pronounced bandwidth enhancement.

Although the bandwidth was significantly increased with the addition of movable masses, the peak V_pp_ was significantly reduced, which would result in reduced power. The preceding conference paper demonstrated a reduction in power of 60% [42]. In general, movable mass power measurements have demonstrated lower power reductions when compared to other methods such as duffing resonators [33,34,42]. Although the peak power was reduced, the power at frequencies off the original RF were increased; therefore the amount of useable energy was similar but more distributed, which is beneficial for practical applications, but there will always be a tradeoff between power and bandwidth. The reduction in voltage/power was caused by the movable masses causing the cantilever to go in and out of resonance during operation, as the outputs are not a single value but an average over a 5 s window.

The choice of movable mass material also significantly influences the bandwidth response. Tungsten masses produced higher bandwidth values especially at higher acceleration compared to Teflon masses. This behavior was attributed to the higher density of tungsten, which increases inertial forces and enhances impact-driven nonlinear interactions. For example, at 1 g, the 2C tungsten configuration achieved a bandwidth of 72 Hz, whereas the corresponding Teflon configuration had a bandwidth of 14.1 Hz. Although the bandwidth increased the voltage output decreased significantly but the overall useable voltage (area under the curve) was significantly higher with the movable masses.

**Figure 9:**
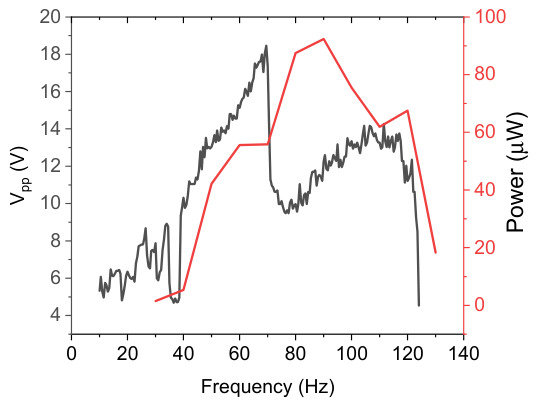
Experimental results of open-circuit voltage and power measurements using a 2-chamber system with tungsten spheres at 1 g acceleration.

The above figures were all open-circuit measurements. A potentiometer connected in parallel was used to manually match the impedance to determine the impact on bandwidth on power measurements. Figure 9 illustrates the results compared to the open-circuit measurements. The open-circuit voltage measurement had FWHM of 72 Hz whereas the power measurement had a FWHM of 69 Hz. This represents a <5% difference in bandwidth, which is similar to what previous research demonstrated [42].

## 4. Conclusions

Overall, this work demonstrates that bandwidth enhancement in the proposed macro-scale system was primarily driven by the introduction of movable masses and the resulting nonlinear dynamic effects. Baseline and fixed-mass configurations remain limited to bandwidths on the order of 5–8 Hz (<10% of RF), whereas free-mass configurations achieved bandwidths as high as 72 Hz, corresponding to an increase of over 1400%. Previous studies investigated a single chamber with movable internal masses. This study determined that increasing the number of chambers can enhance the bandwidth as most movable masses were unable to move the entire distance so having a multi-chamber system limits the masses movements to have a more controlled CoG change. A single chamber could result in a larger change in CoG but the problem was that the movable mass does not have time to roll from one end to the other. However, a multiple-chamber system designed to allow full movement of the movable mass under specific conditions (accelerations and frequencies) could be more effective method at increasing the bandwidth. Higher accelerations and lower frequency applications would benefit from longer chambers, whereas lower accelerations and high frequency applications would benefit from shorter chambers, as these would maximize CoG changes. In addition, having multiple masses in the system can increase the voltage output. These findings demonstrate the effectiveness of the proposed multi-chamber proof-mass design in significantly broadening the operational bandwidth of PEHs while maintaining a passive and mechanically simple architecture by combining two movable mass methods. One significant advantage of movable mass methods was that the output voltage was not dependent on the frequency sweep. Previous results along with results in this study indicate that the output values were similar regardless of the direction of sweeping the frequency. Overall, by optimizing the proof mass, cantilever dynamics, and movable masses, a system could be designed to enhance bandwidth for a particular application, which could be a viable solution to solving the bandwidth issues for high acceleration low frequency applications.

## Figures and Tables

**Table 1:** 3D printed proof-mass model specifications including number of chambers, internal chamber clearance, and proof mass (without movable component).

Model	Number of Chambers	Maximum Movable Mass Distance (mm)	Proof Mass (mg)
1C	1	24	1078
2C	2	8.75	1090
3C	3	3.67	1141
4C	4	1.12	1253

**Table 2:** Proof-mass model mass related configuration specifications including added spacer mass for each configuration and resulting shift in mass.

Model	Spacer Mass (mg)	Tungsten Mass (mg)	Teflon Mass (mg)	Expected Mass Shift Range (%)
1C	21	956	122	0.5–0.6
2C	42	1912	244	0.8–1.2
3C	63	2868	366	1.0–1.7
4C	84	3824	488	1.2–2.1

**Table 3:** FWHM results for all proof-mass models, mass configurations, and acceleration levels.

Model	Mass Type	FWHM Results (Hz)			
		0.5 g		1 g	
		Fixed-Mass	Free-Mass	Fixed-Mass	Free-Mass
1C	Baseline	5.6		8.2	
	Tungsten	6	10.9	8	64
	Teflon	6.1	14.5	8.33	24.1
2C	Baseline	5.8		8.3	
	Tungsten	4.5	31.1	4.8	72
	Teflon	5.9	14.2	8.2	14.1
3C	Baseline	5.6		7	
	Tungsten	4.8	35.7	5.1	36.7
	Teflon	6.2	22.7	8.45	19.4
4C	Baseline	5.8		7.5	
	Tungsten	4.1	35.1	4.9	40.4
	Teflon	6.5	25.8	8.1	20.5

## Data Availability

Data is contained within the article.

## Abbreviations

The following abbreviations are used in this manuscript:

PEH	Piezoelectric energy harvesters
RF	Resonance frequency
CoG	Center-of-gravity
FWHM	Full-width half max
